# CAN008 prolongs overall survival in patients with newly diagnosed GBM characterized by high tumor mutational burden

**DOI:** 10.1016/j.bj.2023.100660

**Published:** 2023-09-21

**Authors:** Ian Yi-Feng Chang, Hong-Chieh Tsai, Chia-Hua Chen, Hsiu-Chi Chen, Chia-Wen Huang, Gerald F. Cox, Fang-Min Huang, You-Yu Lin, Ko-Ting Chen, Ya-Jui Lin, Kuo-Chen Wei

**Affiliations:** a Molecular Medicine Research Center, Chang Gung University, Taoyuan, Taiwan; b School of Medicine, Chang Gung University, Taoyuan, Taiwan; c School of Traditional Chinese Medicine, Chang Gung University, Taoyuan, Taiwan; d Department of Neurosurgery, Chang Gung Memorial Hospital, Linkou Medical Center, Taoyuan, Taiwan; e Neuroscience Research Center, Chang Gung Memorial Hospital, Linkou Medical Center, Taoyuan, Taiwan; f Genome and Systems Biology Degree Program, Academia Sinica and National Taiwan University, Taipei; g Department of Neurosurgery, New Taipei Municipal TuCheng Hospital, New Taipei City, Taiwan; h CANbridge Pharmaceuticals, Burlington, MA, USA; i CANbridge Pharmaceuticals Inc., Shanghai, China

**Keywords:** Asunercept, CAN008, GBM, Tumor mutational burden, Immunotherapy

## Abstract

**Background:**

A previous phase 1 dose-escalation study in Taiwan indicated CAN008 (asunercept) with standard concurrent chemoradiotherapy (CCRT) improved progression-free survival (PFS) in newly diagnosed glioblastoma (GBM) patients. This study evaluates the efficacy of CAN008 in promoting overall survival (OS) and identifies genetic alterations associated with treatment responses.

**Methods:**

We compared OS of 5-year follow-ups from 9 evaluable CAN008 cohort patients (6 received high-dose and 3 received low-dose) to a historical Taiwanese GBM cohort with 164 newly diagnosed patients. CAN008 treatment response-associated genetic alterations were identified by whole-exome sequencing and comparing variant differences between response groups. Associations among patient survival, tumor mutational burden (TMB), and genetic alterations were analyzed using CAN008 cohort and TCGA-GBM dataset.

**Results:**

OS for high-dose CAN008 patients at 2 and 5 years was 83% and 67%, respectively, and 40.1% and 8.8% for the historical GBM cohort, respectively. Better OS was observed in the high-dose CAN008 cohort (without reaching the median survival) than the historical GBM cohort (median OS: 20 months; *p* = 0.0103). Five high-dose CAN008 patients were divided into good and poor response groups based on their PFS. A higher variant count and TMB were observed in good response patients, whereas no significant association was observed between TMB and patient survival in the newly diagnosed TCGA-GBM dataset, suggesting TMB may modulate patient CAN008 response.

**Conclusion:**

CAN008 combined with standard CCRT treatment prolonged the PFS and OS of newly diagnosed GBM patients compared to standard therapy alone. Higher treatment efficacy was associated with higher TMB.

## Highlights

• CAN008 (asunercept) combined with CCRT prolonged the survival of newly diagnosed GBM patients compared to standard therapy.
• Higher treatment efficacy of CAN008 was associated with a higher tumor mutational burden.
• Mutations of *DMD*, *DNAH12*, *HUWE1*, *MYCBP2*, *SLC11A2*, and *ZFC3H1* were associated with CAN008 treatment response.

## Introduction

Glioblastoma (GBM) is a common malignant brain tumor derived from glial cells, and it accounted for 49.1% of malignant tumors in the central nervous system in the United States from 2014 to 2018 [1]. The conventional treatment for GBM is surgical resection, followed by radiochemotherapy for 6 weeks and adjuvant temozolomide for 6 months. However, the therapeutic effect is poor, as the five-year survival rate of GBM patients is only 5% [1]. Recent studies aiming to improve treatment efficiency have focused on enhancing drug delivery across the blood‒brain barrier with methods including nanoparticle encapsulation and focused ultrasound, as well as on studying the molecular mechanisms related to tumorigenesis and progression to find effective targets for specific therapies, such as epidermal growth factor receptor, vascular endothelial growth factor, and neurotrophic tyrosine receptor kinase fusions [[2], [3], [4], [5]]. In addition, the cellular heterogeneity and stemness characteristics of GBM facilitate tumor resistance to standard therapy and result in rapid recurrence, and the diffusive nature of GBM also hinders total surgical removal and emphasizes the importance of local treatment [6,7]. Recent advancements toward understanding the GBM tumor immune microenvironment have led to the proposed potential of immunotherapy targeting the crosstalk between immune and tumor cells, such as CAR T-cell therapy, dendritic cell therapy, and immune checkpoint blockade [8].

CD95, encoded by the *FAS* gene, belongs to the death receptor family, and upon binding to its cognate ligand, CD95L, it triggers a signal that results in programmed cell death, which is critical for immune-mediated elimination of virus-infected or transformed cells [9,10]. In the past two decades, involvement of the CD95/CD95L system in different biological processes has been discovered in several diseases, including cancers [11]. Constitutive activity of CD95 is required for optimal growth in cancer cells, and loss of CD95 in mouse models of ovarian and liver cancers resulted in reduced tumor size [12]. Stimulation of CD95 by soluble CD95L induces calcium-dependent activation of the c-yes/PLCγ1/PI3K/Akt pathway that promotes cell migration in triple-negative breast cancer [13]. In GBM, *FAS* expression is upregulated in tumors compared to non-tumor tissues, and lower *FAS* expression is correlated with better overall survival (OS) [14]. Additionally, activating CD95 by ligand binding enhanced cell invasion through a PI3K/glycogen synthase kinase 3-beta pathway/matrix metalloproteinase-mediated pathway in glioblastoma cells, and blockade of CD95-activated signaling suppressed glioma invasion in an *in vivo* mouse model [15]. In contrast, knockdown of the *FASLG* gene, which encodes CD95L, reduced cell invasiveness without significantly altering cell growth in GBM cell lines [16].

Asunercept, the trade name for APG101 (manufactured by Rentschler Biopharma SE, Germany, for Apogenix AG) and CAN008 (manufactured by WuXi Biologics, China, for CANbridge Pharmaceuticals), is a recombinant CD95L-binding Fc-fusion protein that consists of the extracellular domain of human Fas/CD95 that is linked to the Fc domain of human IgG1 [17]. It selectively binds to CD95L and blocks the CD95L-Fas/CD95 interaction as well as activation of the subsequent signaling pathway. APG101 suppressed CD95L-induced cell invasion without altering cell viability in GBM cell lines, reduced tumor growth and matrix metalloproteinase (MMP) activity in combination with radiotherapy in intracranial glioma-bearing mouse models and resulted in longer mouse survival than radiotherapy alone [16,18]. A controlled-randomized phase 2 trial with APG101 in 84 recurrent GBM patients showed a reduction in tumor growth and an improvement in progression-free survival (PFS) in patients with secondary radiotherapy combined with weekly APG101 treatment compared to radiotherapy alone [19]. We previously conducted a phase 1 open-label, dose-escalation trial with CAN008 combined with standard concurrent chemoradiotherapy in 10 Asian patients with newly diagnosed GBM [20]. The results showed that the treatment was safe, and there was a higher 6-month progression-free survival (PFS6) rate in patients treated with 400 mg/week CAN008 than in patients treated with 200 mg/week CAN008, suggesting the potential efficacy of CAN008 in treating newly diagnosed GBM patients.

As an improvement in PFS by 400 mg/week CAN008 treatment has been demonstrated in previous reports [20], this study aimed to examine the effect of CAN008 on overall survival of GBM patients and to compare the survival of CAN008 patients to a historical cohort of Taiwanese GBM patients who received standard treatment. All patients, the CAN008 group and the historical GBM group, were treated at the same clinical center in Linkou Chang Gung Memorial Hospital, Taiwan. Previous results of asunercept have shown that low CpG2 methylation of the *CD95L* promoter was associated with greater asunercept responses and improved treatment outcome in both newly diagnosed and recurrent GBM patients, therefore representing a potential biomarker for indicating the asunercept treatment outcome [19,20]. In addition, the study found an association between a good CAN008 treatment response and a high rate of somatic mutations and tumor mutational burden (TMB), suggesting that the number of genetic alterations may be a potential predictor of the CAN008 treatment response and could help guide the future applications of CAN008 (asunercept) in cancer treatment.

## Materials and methods

### Patients

**Fig. 1:**
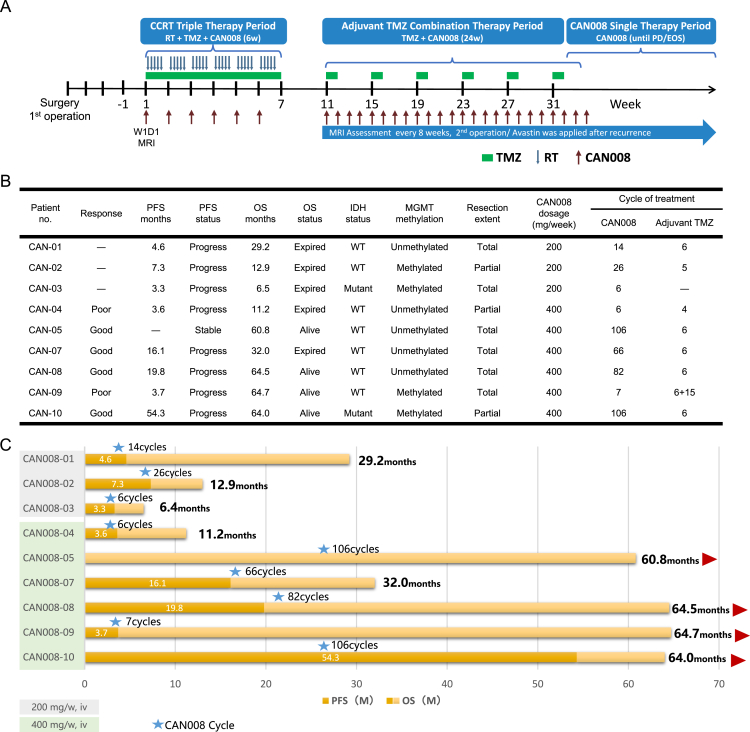
The CAN008 treatment protocol and basic treatment information for 9 patients. (**A)** Treatment protocol. Patients were treated with CAN008 weekly until the disease progressed or the end of the study. (**B)** A table of patient survival and treatment information. The CAN-09 patient was treated with conventional CCRT treatment with CAN008 and adjuvant TMZ with CAN008 for 6 weeks from January 16, 2018 to June 5, 2018. After the MRI images indicated the recurrent GBM in 2021, the patient started treatment with TMZ alone again from May 26, 2021 to June 29, 2022 for 15 weeks. (**C)** The bar chart represents patient survival (in months) and CAN008 treatment cycles. CCRT: concomitant chemoradiotherapy; TMZ: temozolomide; EOS: end of study; RT: radiation therapy; PD: progressive disease; PFS: progression-free survival; OS: overall survival; WT: wild-type IDH.

CAN008 cohort patients were recruited in a phase 1/2 open-label trial (NCT02853565) that started in August 2016 and was completed in September 2018. Ten patients were enrolled in the trial, and the study design, safety report, and PFS were reported [20]. Nine evaluable patients (3 received 200 mg/week and 6 received 400 mg/week CAN008 treatment) were followed-up in Linkou Chang Gung Memorial Hospital, Taiwan, and were included in this study. The treatment protocol and basic patient information are listed in [Fig. 1A, B, and 1C]. The historical GBM cohort in this study contained 164 newly diagnosed GBM patients receiving brain tumor surgical resection and concomitant chemoradiotherapy treatment (CCRT) at Linkou Chang Gung Memorial Hospital, Taiwan, between May 2003 and September 2020, followed by standard of care. Patient sample collection and usage were approved by the Chang Gung Medical Foundation Institutional Review Board (201502656B0 and 202101628B0); written informed consent was obtained from patients prior to sample collection. The cohort consisted of 115 male and 54 female patients with a median age at diagnosis of 61 years (range, 21–85 years) and a median follow-up duration after diagnosis of 417 days (range, 51–2250 days). Tumor grading and pathology was determined based on morphology, arrangement of cells in tumors, IDH status, and 1p/19q codeletion according to the 2016 CNS WHO. All patients in this study, the CAN008 group and the historical control group, were treated at the same clinical center in Linkou Chang Gung Memorial Hospital, Taiwan. Patient survivals were defined as the time from the date of first diagnosis of GBM to the date of patient death for OS or the date of first radiological evidence of progression or death due to any cause for PFS. The Magnetic Resonance Imaging (MRI) images were evaluated according to RANO by radiologists.

### Whole-exome sequencing (WES)

DNA was extracted from homogenized tumors and paired peripheral blood mononuclear cells (PBMCs) using the Gentra Puregene Tissue Kit (Qiagen) according to the manufacturer's instructions. For patient CAN-08, the genomic DNA of the tumor was extracted from FFPE samples using the column-based method (iCatcher FFPE Tissue DNA Kit, CatchGene). The degradation degree and potential contamination of the DNA were monitored on 1% agarose gels. DNA quantification was performed using a Qubit® dsDNA Assay Kit in a Qubit® 4.0 Fluorometer (Life Technologies, CA, USA). Sequencing libraries were prepared from 1 μg of DNA per sample and were generated using Agilent SureSelect Human All Exome V6 (Agilent, USA) following the manufacturer's recommendations. The library quality was assessed on a Qubit 4.0 Fluorometer and Agilent Bioanalyzer system. Library sequencing was performed on an Illumina NovaSeq 6000 platform, which generated paired 150‐bp reads.

### WES data preprocessing

The Genome Analysis Toolkit (GATK, version 4.2.6.1) best practice guideline (https://gatk.broadinstitute.org) was used to process all WES files into analysis-ready BAM files, and the GATK bundle resources were downloaded from the Broad Institute. Paired-end reads were aligned to the human reference genome (hg38) using BWA-mem (version 0.7.17). Read group information was added to SAM files, which were then converted to BAM files by GATK MergeSamFiles. The aligned reads were subsequently processed with GATK MarkDuplicatesSpark, BaseRecalibrator, and ApplyBQSR. The identity of paired BAM files from one patient was examined by calculating the pairwise correlations of variant allele fractions (VAFs) at known SNP sites and classifying the VAFs by NGSCheckmate [21].

### Somatic mutation detection

Variant calling for somatic single nucleotide variants (SNVs) and small insertions/deletions (INDELs) was performed using GATK MuTect2 and VarScan2. GATK ContEst was used to estimate the cross-individual contamination. The Mutect2 read orientation artifacts workflow was adopted to eliminate potential library preparation and sequencing artifacts. The variants were annotated using ANNOVAR with GENCODE v32. Maftools was used to convert ANNOVAR output information into MAF format for variant analyses. The TMB in this article refers to the number of nonsynonymous somatic variants (SNVs/INDELs) of a cancer cell. To compare the TMB between different patient cohorts, the values of TMB were normalized into number of mutations per megabase by dividing the target size of WES kit.

$$Normalized\;TMB=\frac{\text{nonsynonymous}\;\text{somatic}\;\text{variants}}{\text{target}\;\text{size}}$$

Nonsynonymous somatic variants: SNVs/INDELs.

Target size: 60 Mb in Our cohort (Agilent SureSelect V6) and 35.8 Mb in TCGA cohort.

WES was performed in our patient cohort using Agilent SureSelect V6, while there were 8 WES kits used in TCGA cohorts (https://www.ncbi.nlm.nih.gov/pmc/articles/PMC6169918/). The capture sizes are 60 Mb in our cohort and 35.8 Mb assumed by maftools in TCGA cohorts (https://rdrr.io/bioc/maftools/man/tcgaCompare.html).

### TCGA-GBM dataset

The somatic SNVs and INDELs of TCGA cohort determined by MuTect2 and VarScan2 were downloaded from the Genomic Data Commons Data Portal, and patient clinical information was obtained from cBioPortal. The GBM cohort of The Cancer Genome Atlas (TCGA-GBM) includes 366 newly diagnosed GBM patients with complete SNV, of whom 363 have complete clinical information with a male to female ratio of 225:138 and a median age at diagnosis of 61 years (range, 21–89 years). To compare the TMB level between patients with or without the mutated genes, the tables of the TMB counts and somatic mutations were downloaded from cBioPortal and processed using the “maftools” package.

### Statistical analysis

The Wilcoxon rank sum test was performed to determine the significant differences between two independent groups. Survival probabilities were estimated using the Kaplan‒Meier method; the differences between groups were analyzed using log-rank tests. The Kaplan‒Meier curves, log-rank test *p* value, and median survival time were calculated and presented by the R packages “survival” and “survminer”. All statistical tests were two-tailed; *p* < 0.05 was considered statistically significant.

## Results

### CAN008 treatment outcomes in newly diagnosed GBM patients in Taiwan

**Fig. 2:**
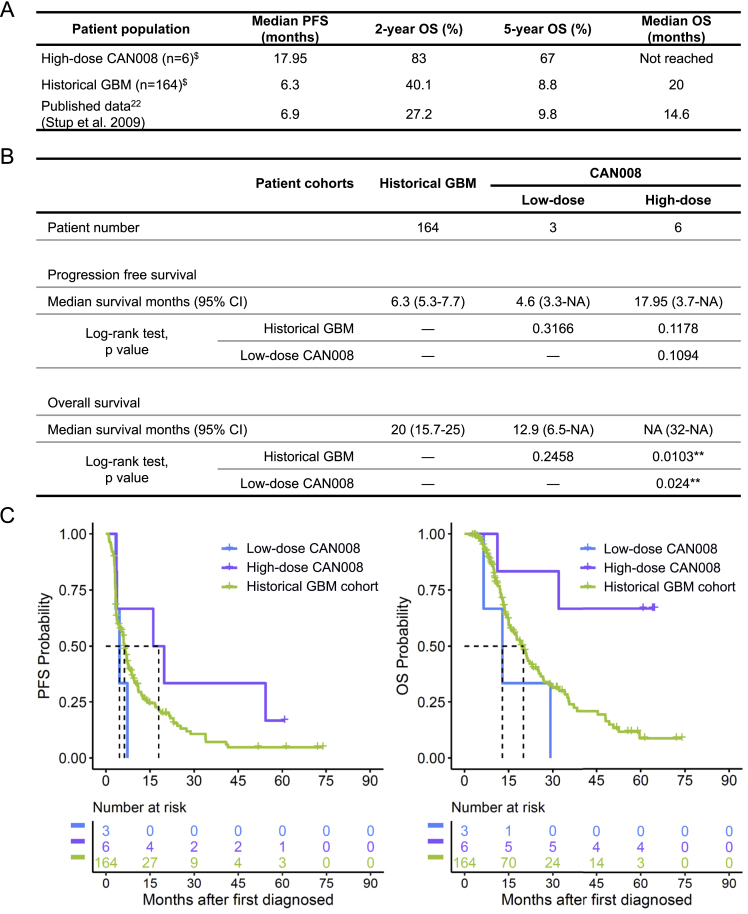
Treatment outcomes of CAN008 with dosage and survival information of GBM patients in historical GBM cohort and published data. (**A)** Survival information of newly diagnosed GBM patients in high-dose CAN008 cohort, historical GBM cohort, and published data. ^$^Patients treated at Linkou Chang Gung Memorial Hospital, Taiwan. (**B)** Median survival months and treatment efficacy among cohorts. CI: confidence interval. NA: not available due to not enough events to estimate the upper confidence. ∗*p* < 0.05; ∗∗*p* < 0.01. (**C)** Kaplan‒Meier survival curves of the historical GBM cohort and CAN008 cohorts with different dosages in Taiwan.

Nine of the 10 patients with newly diagnosed GBM who received CAN008 in a previous phase 1/2 clinical trial were treated in Linkou Chang Gung Memorial Hospital and monitored for at least 5 years postsurgery. Only patients treated at this center were considered for the analysis to eliminate center to center variations. A historical Taiwanese GBM cohort consisting of 164 newly diagnosed GBM patients served as a reference of treatment outcomes of patients treated with standard therapy at this center. Survival results of the historical GBM cohort are well aligned with published data [Fig. 2A] [22]. Three patients received 200 mg/week CAN008 (low-dose group), and six were treated with 400 mg/week CAN008 (high-dose group). The median PFS in the high-dose group (17.95 months, 95% CI: 3.7-NA months) was longer than that of the historical GBM cohort (6.3 months, 95% CI: 5.3–7.7 months, [Fig. 2B]). However, the difference in the corresponding Kaplan‒Meier curves was not statistically significant (*p* = 0.1178, log-rank test), most likely due to the small size (n = 6) of the CAN008 high-dose group [Fig. 2C]. The OS of the high-dose group did not reach the median survival within the 5-year follow-up, whereas the median OS of the historical GBM cohort and low-dose group were 20 (95% CI: 15.7−25) and 12.9 (95% CI: 6.5−NA) months, respectively [Fig. 2B]. A highly statistical significant difference in Kaplan‒Meier curves for OS was observed between the high-dose group and the historical GBM cohort (*p* = 0.0103, log-rank test). The high-dose group had a higher OS probability than the historical GBM cohort, with a 2-year OS of 83% (95% CI: 0.583–1) versus 40.1% (95% CI: 0.319–0.505) and a 5-year OS of 67% (95% CI: 0.379–1) versus 8.8% (95% CI: 0.038–0.206), respectively [Fig. 2A]. The results indicate a marked improvement in the OS of newly diagnosed GBM patients after the addition of high-dose CAN008 to standard treatment.

**Fig. 3:**
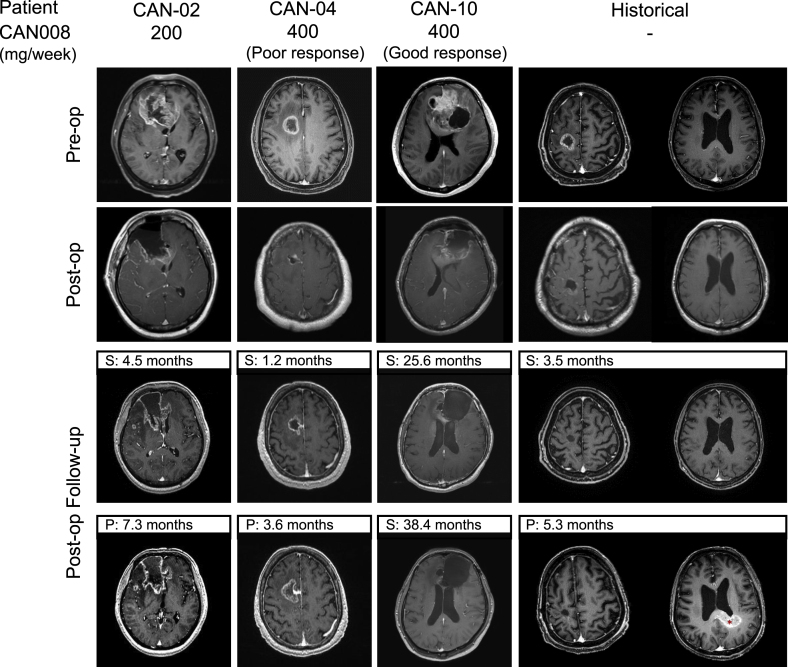
Representative MRI imagines of patients in low-dose, poor response, and good response groups and the historical GBM cohort. Patient CAN-02 in low-dose group was treated with 200 mg/week CAN008. Patient CAN-04 and patient CAN-10 were treated with 400 mg/week CAN008 with different treatment response as poor and good, respectively. Red asterisk: enhancing lesion of the progressed tumor. pre-op: preoperation, post-op: post operation, S: stable disease, P: progression.

Patients in high-dose group were divided into the poor and good response groups if their PFS was shorter or longer than the 6.3-month median PFS of the historical GBM cohort, respectively [Fig. 1B]. The representative MRI images of patients in the low-dose group, poor and good response groups, and the historical GBM group were shown in [Fig. 3]. Patients CAN-02, CAN-04, and CAN-10 all underwent partial surgical resection and have different outcomes of the disease. Patient CAN-02 in the low-dose group showed a stable disease after surgery for 4.5 months, and the enlargements of enhancing lesion at the right periventricular region and the right frontal distant enhancing nodule were observed post-op for 7.3 months. In the high-dose group, the tumor progressed soon after 3.6 months post-op in patient CAN-04, whereas patient CAN-10 showed stable disease after 38.4 months post-op follow-up. The representative historical GBM patient underwent total resection. The disease was stable for the first 3.5 months post-op but progressed after 5.3 months post-op in occipital region, whereas no enhancement was observed in frontal lobe where the tumor primarily occurred.

### Association between TMB and the CAN008 treatment response

**Fig. 4:**
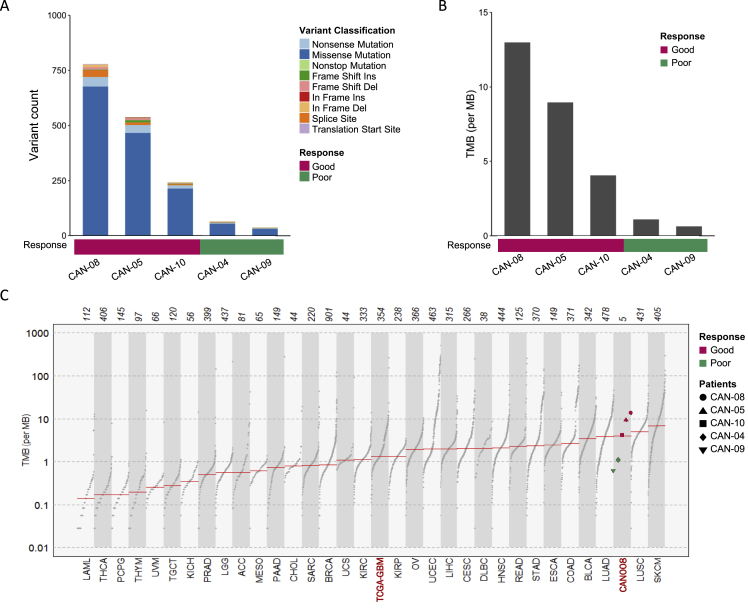
Somatic mutation and TMB in the CAN008 cohort. (**A)** Variant counts and (**B)** TMB in each patient of the CAN008 cohort. (**C)** Comparison of TMB measured in mutations per megabase (Mb) of DNA in the CAN008 and 33 TCGA datasets. Horizontal lines: median frequencies of TMB in each dataset.

To identify potential genetic alterations contributing toward the differential treatment response of CAN008, WES was performed on five pairs of tumors and PBMC specimens from patients receiving high-dose CAN008, including CAN-04, CAN-05, CAN-08, CAN-09, and CAN-10. The specimen from patient CAN-07 was excluded from all subsequent analyses due to marginal sampling with histology that did not match the main pathology. Patients with a good response (CAN-05, CAN-08, and CAN-10) had more somatic mutations and structural variants detected than those of patients with a poor response (CAN-04 and CAN-09; [Fig. 4A]). Missense mutations were the most abundant alterations, accounting for approximately 85% of all variants, followed by nonsense mutations with approximately 5% in each sample. The TMBs, calculated from all tumor variants, were also higher in patients with a good response than in patients with a poor response to CAN008 [Fig. 4B]. The TMBs of all three patients with a good response were also higher than the median TMB of the TCGA-GBM cohort (N = 366), whereas the two poor-response patients had TMBs lower than the median TMB of the TCGA-GBM cohort [Fig. 4C]. WES was also performed in low-dose CAN008 patients, but the associations among patient response, survival, and TMB were inconclusive because the treatment efficacy of low-dose CAN008 was undefined [Supplementary Fig. 1]

**Fig. 5:**
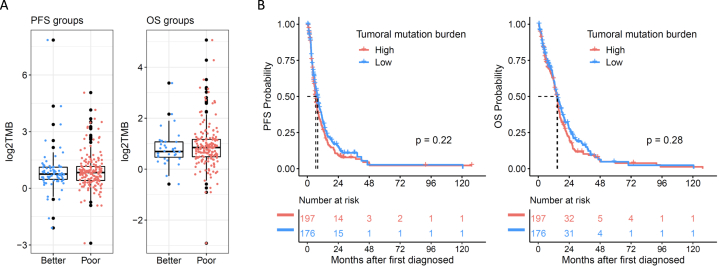
Correlation of patient survival and TMB in the TCGA-GBM dataset. (**A)** Comparison of TMB in patients with better and poorer survival in the PFS or OS groups. (**B)** Kaplan‒Meier survival curves of patients with high and low TMB. p, log-rank test, *p* value.

The TCGA-GBM dataset (366-TCGA-GBM cohort) was examined to test for an association between TMB and PFS/OS in the absence of CAN008. Newly diagnosed GBM patients were divided into two groups according to their survivals or TMBs. Patients who died or had tumor progression before the median survival time were categorized as the poor survival group, whereas patients who lived or stayed progression-free longer than double the median survival time were categorized as the better survival group. Patients with their TMBs equal or higher than median TMB of this cohort were grouped to TMB high group, while others were grouped to TMB low group. No significant difference in TMB level was observed between poor and better survival patients in both the PFS and OS groups [Fig. 5A], and similarly, no significant survival difference was observed between patients with high and low TMB [Fig. 5B]. The results indicated a potential association of TMB with survival that may result from the treatment response of CAN008.

### Differential genetic variants between the good and poor response groups

**Fig. 6:**
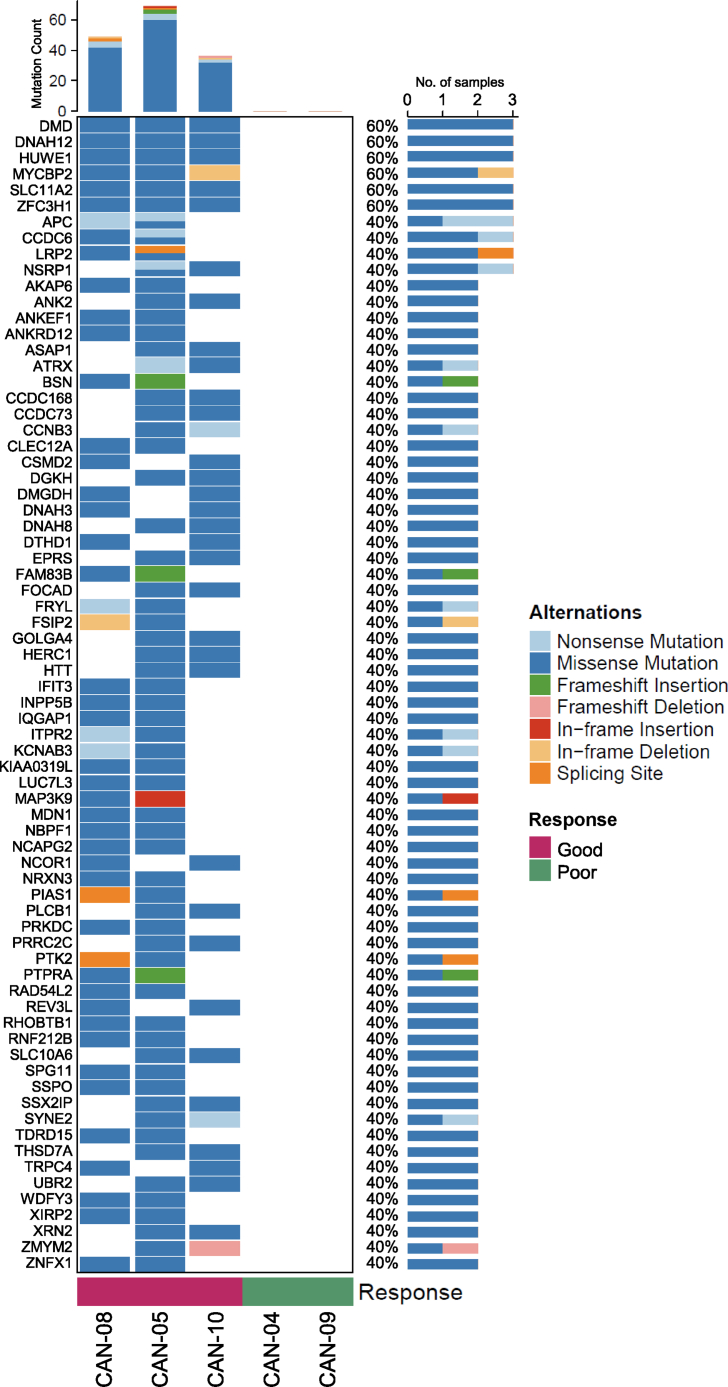
Oncoplot of gene alterations in patients with a good response to CAN008.

**Fig. 7:**
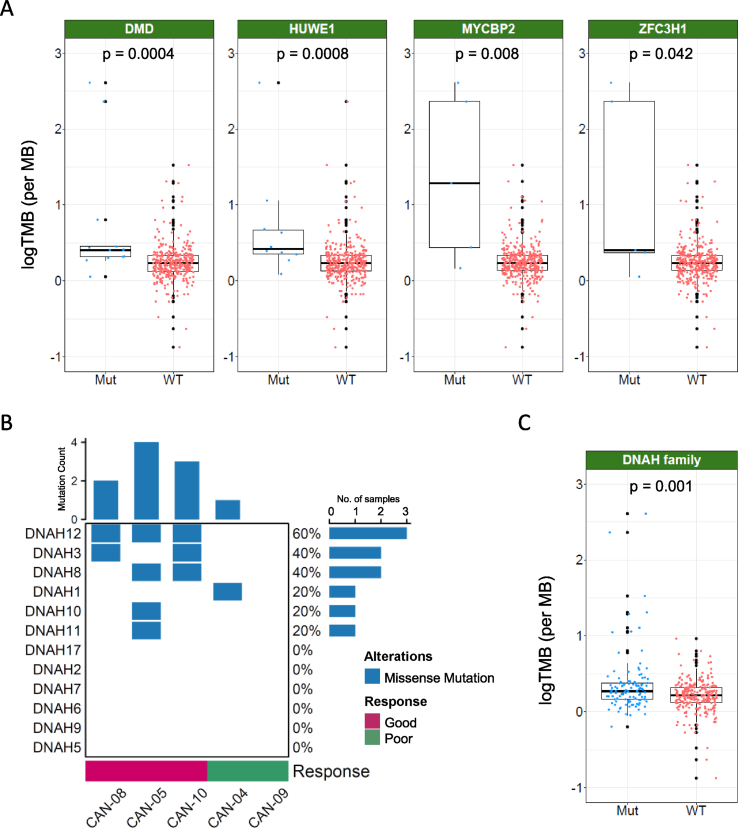
Correlation of TMB with gene alterations associated with a good CAN008 response in the newly diagnosed TCGA-GBM cohort. (**A)** TMBs in TCGA-GBM patients with wild-type or mutant *DMD*, *HUWE1*, *MYCBP2*, and *ZFC3H1*. (**B)***DNAH* mutations in five patients treated with high-dose CAN008. (**C)** TMBs in TCGA-GBM patients with or without *DNAH* mutations. Mut: mutant; WT: wild type.

As TMB is a collection of various types of genetic alterations in tumors, we tried to find the genetically altered genes that were correlated with the treatment response of CAN008 by comparing the mutated genes between the two response groups. Mutated genes that were detected in more than one sample of a single response group but not in the other group are listed in [Fig. 6]. A total of 72 mutated genes were identified in the good response group, whereas none were identified in the poor response group. However, Gene Set Enrichment Analysis (GSEA) of the 72 mutated genes with the KEGG, Reactome, BioCarta, PID, and WikiPathways databases revealed no significantly enriched canonical signaling pathway. Six genes, namely, *DMD*, *DNAH12*, *HUWE1*, *MYCBP2*, *SLC11A2*, and *ZFC3H1*, were mutated in all patients with a good CAN008 response, and their mutation frequencies in the 366-TCGA-GBM cohort were 3.6% (13/366), 0.5% (2/366), 2.2% (8/366), 1.1% (4/366), 0.3% (1/366), and 1.4% (5/366), respectively. Furthermore, the *DMD* and *HUWE1* mutation rates ranked 60 and 250, respectively, among 12,601 mutated genes in the 366-TCGA-GBM cohort (median mutation rate 0.55%). Moreover, higher TMB counts were observed in patients with mutated *DMD* (Wilcoxon rank sum test, *p* < 0.0001), *HUWE1* (*p* = 0.001), *MYCBP2* (*p* = 0.003), and *ZFC3H1* (*p* = 0.019) compared to patients with wild-type genes [Fig. 7A]. In a previous gastric cancer study, a significantly higher number of mutations was observed in *DNAH*-mutated patients than in *DNAH* wild-type patients [23], which suggests an association between the *DNAH* family and TMB. Here, we report that 6 of the 12 *DNAH* family genes were mutated in the CAN008 patient cohort, and *DNAH3*, *DNAH8*, *DNAH10*, *DNAH11*, and *DNAH12* were only mutated in patients with a good response, whereas *DNAH1* was mutated in patient CAN-04 with a poor response [Fig. 7B]. In the 366-TCGA-GBM cohort, mutated *DNAH*s were observed in 89 patients (24.32%), and the TMB was correlated with the status of the *DNAH* family, as patients with mutated *DNAH* had a higher TMB than that of patients with wild-type *DNAH* [Fig. 7C]. The results indicate that several mutated genes in patients with a good response were associated with a higher TMB and may independently contribute to the CAN008 treatment difference. However, the exact functional changes in the mutated genes, their influence on CAN008 treatment response, and the involved molecular mechanisms require further experimental effort to clarify.

### CAN008 treatment response and antitumor immunology

TMB has been reported to correlate to immunotherapy response that higher TMB is associated with better treatment response. The CAN008 treatment response in this study is also correlated to TMB, therefore we tried to investigate the association between antitumor immunology and CAN008 treatment response in the CAN008 patient cohort. The expression of immune checkpoint inhibitors, PD-1 and PD-L1, and the T-cell receptor inhibitory molecule, CD5, were measured by immunohistochemistry staining to evaluate the antitumor immunoactivity in tumors. No PD-1 staining was found in most of the patients, and there were only a few sporadically positive cells in patients CAN-05 and CAN-08 (data not shown). The PD-L1 and CD5 staining was observed in most of the patients, except for patients CAN-03 and CAN-04. The intensity and localization of the PD-L1 staining in tumors varied among patients. Strong intercellular substance staining was observed in patients CAN-04, CAN-05, CAN-08, and CAN-09, whereas strong cellular staining could be observed in patients CAN-02, CAN-05, and CAN-10 [Supplementary Fig. 2A]. The CD5 staining was found in most of the patients, and many CD5-positive cells were found around blood vessels. Patients who were positive of PD-L1 staining had CD5-positive cells in their tumors [Supplementary Fig. 2B]. Patients in the good response group were all positive of both markers, whereas one out of two patients in the poor response group had double positive staining. Besides, patient CAN-02 in the low-dose group, who expressed CD5 and high PD-L1, had a PFS longer than the median PFS of the historical GBM cohort. However, we cannot draw any conclusion of the association between antitumor immunoactivity and CAN008 treatment response in this study, due to the small sample size.

## Discussion

Activation of the CD95/CD95L system in cancer may result in drastically different biological outcomes, such as apoptosis, sustained cell growth, and enhanced cell invasiveness, which depend on the involved signaling pathways, interacting cell types, and tumor microenvironment [10,11]. Although CD95 was first known as a prototypic death receptor that was activated by binding to CD95L and triggers programmed cell death, the CD95/CD95L system is also involved non-apoptotic signaling. The incomplete activation of CD96 downstream signaling cascades may also lead to tumor-promoting effects through activation of MAPK, NF-κB, and PI3K pathways, which contribute to cell proliferation, survival, and migration. Additionally, as CD95 and CD95L are expressed in various cell types, including tumor cells and immune cells, their functions involve tumor-immune communication and contribute to immune regulation, such as inflammation. Asunercept (APG101/CAN008), an inhibitor of the CD95/CD95L signaling pathway, effectively suppressed the CD95L-increased cell invasion in GBM cells and reduced tumor invasion into surrounding tissues in intracranial xenograft mouse models, and it prolonged mouse survival [16,18]. The results of this study combined with previous clinical trial studies indicate that asunercept could effectively increase PFS and OS in recurrent GBM and in newly diagnosed GBM patients, which implies the great potential of asunercept as a novel therapy with good efficacy for treating GBM patients with different statuses.

Although the phase 2 clinical trial of asunercept targeted recurrent GBM and our previous phase 1/2 clinical trial recruited newly diagnosed GBM patients, the results of both clinical trials consistently demonstrated that asunercept improved the survival of GBM patients [19,20]. Recurrent GBM currently lacks a standard treatment partly because of the contribution of increased somatic mutations, stemness characteristics, and enhanced malignant phenotypes of tumor cells in recurrent tumors that result in tumor resistance to radiotherapy and chemotherapy with temozolomide. Therefore, the treatment of recurrent GBM is often concurrent with bevacizumab or other therapies within clinical trials based on the patient and tumor condition. In the asunercept (=APG101) clinical trial against recurrent GBM, asunercept was added to the treatment concurrent with reirradiation after tumor recurrence [19]. The results showed that combined therapy significantly improved PFS compared to radiation therapy alone. Recently, the addition of targeted therapy to the first-line treatment of newly diagnosed GBM was proposed by physicians and clinical researchers with the belief that early treatment could more effectively suppress tumor progression and improve patient prognosis. In the CAN008 clinical trial for newly diagnosed GBM, CAN008 was used with CCRT as the first-line treatment, and the OS of patients with high-dose CAN008 was significantly improved compared to patients with CCRT alone [20]. Although the number of cases in this clinical trial was small, the trial results suggest the potential of CAN008 as a first-line treatment in newly diagnosed GBM with good treatment efficacy.

Although studies on asunercept function in GBM mainly suggest that it inhibits cell/tumor invasiveness and prolongs glioma-bearing mouse survival, the asunercept-targeted CD95/CD95L signaling pathway is also involved in cancer immune regulation [16,18]. The binding of CD95L expressed by tumor cells or other cells in the tumor microenvironment to CD95-bearing activated T cells or NK cells induces apoptosis of these immune cells. This CD95L mediated elimination of anti-cancer immune cells establishes a barrier towards the immune system and helps tumor cells to escape an immune attack [10,24,25]. Therefore, the binding of asunercept to CD95L may affect immune regulation in the GBM microenvironment, rendering the patient's response to asunercept. A recent study on CD5 indicated that the expression of CD5 on dendritic cells enhanced T cell priming and response to immune checkpoint blockade therapy through generating optimally protective CD5^hi^ T helper and CD8^+^ T cells [26]. Although the 3-evaluable patients in the good response group all expressing PD-L1 and possessing CD5-positive cells in their tumors, the relationship between CAN008 response and the activation of antitumor immunity is still unclear due to the small sample size. More effort is required to reveal the exact molecular mechanism and biological process involved in the asunercept-mediated interaction between tumors and the immune microenvironment.

Methylation at CpG2 in the CD95L promoter region in both newly diagnosed and recurrent GBM as well as CD95L protein expression in newly diagnosed GBM has been indicated to be associated with the treatment efficacy of asunercept [19,20]. In this study, we demonstrated that TMB is another potential selection marker for asunercept treatment in newly diagnosed GBM patients, as a higher TMB was associated with a better CAN008 treatment response. TMB has been extensively described as a predictive biomarker of immunotherapy response [27,28]. The TMB triggers antitumor immune reaction through a way different than PD-1/PD-L1 and CD5 that regulate the immune checkpoint pathway. A higher TMB gives more immunogenic neoantigens that could be recognized by host T cells, resulting in a better response to immunotherapy [29]. The FDA has approved pembrolizumab (Keytruda), a humanized monoclonal antibody against human programmed death receptor-1 (PD-1), for treating solid tumors with high TMB (defined as ≥10 mutations/Mb) [30]. Pembrolizumab blocks the binding of PD-1 to PD-L1 and works as an immune checkpoint inhibitor that promotes the killing of tumor cells by T cells [31].

The correlation of TMB with immunotherapy response has been demonstrated in many studies, and high TMB has been used as a selection marker for immunotherapy. However, whether a single TMB value should be used as the standard for the selection of immunotherapy is still under debate. The efficacy of immunotherapy was correlated not only with TMB but also with the cell composition in the tumor microenvironment as well as the tumor status and immune response. As the methods of TMB measurement vary among studies and the levels of TMB differ among cancers, it is difficult to use a single TMB standard to determine the patient response for different situations, and more extensive studies must be performed to improve the practice of using the TMB as a screening marker for immunotherapy. In this study, we report that patients’ responses to CAN008/asunercept were associated with TMB and with mutations in two genes, *DMD* and *HUWE1*, which had high mutation frequencies in newly diagnosed GBM. The *DMD* gene, which encodes dystrophin, is currently the largest known gene, accounting for 0.08% of the human genome. Mutations in this gene can cause varying degrees of muscular dystrophy and dilated cardiomyopathy, and it has recently been found to be involved in the regulation of cancer progression [32]. The *DMD* splicing isoform Dp71 is a dystrophin protein that is mainly expressed in the brain and functions to maintain the integrity of the blood‒brain barrier [33]. The expression of the Dp71 isoform was observed in normal human astrocyte cell lines, and expression was decreased in GBM cell lines as well as human primary GBM cells [34]. In addition, higher Dp71 expression was associated with a lower Ki67 proliferation index and better prognosis in GBM patients. *DMD* may participate in GBM progression, but the exact role and molecular mechanism are still unclear. *HUWE1* encodes an E3 ubiquitin ligase, and many of its substrates, such as p53, ATM, and CDKN2A, are involved in tumor progression. *HUWE1* was also downregulated in GBM compared to adjacent nontumor tissue, and low expression of HUWE1 was correlated with poor survival in GBM patients [35]. HUWE1 suppressed cell growth and invasion in GBM cells and inhibited HUWE1 expression, prolonging the survival of intracranial tumor-bearing mice. In addition to *DMD* and *HUWE1*, we also found an association of *DNAH* family mutations with TMB, which was in accordance with findings that somatic mutation of the *DNAH* gene family was associated with higher TMB and better chemotherapy treatment response in gastric cancer patients [23]. Since TMB is the product, but not the cause, of genetic alterations in tumors, identifying the molecular mechanisms and specific factors that directly contribute to the difference in the asunercept response may be useful for future selection of asunercept treatment.

We demonstrated in this study that the addition of asunercept (APG101/CAN008) to CCRT in the conventional treatment of newly diagnosed GBM patients could efficiently improve the OS of patients. Patients with a higher TMB had a better response to asunercept treatment, which points toward a potential immunoregulatory role of asunercept function in the GBM microenvironment. The case number in the present study was too small to obtain a statistically reliable conclusion and to perform more detailed analyses. A phase 2 clinical trial was initiated in 2021 and is recruiting newly diagnosed GBM patients in mainland China (www.clinicaltrials.gov, NCT05447195), to gather more molecular profiles and possibly identify the mechanism of the patient response to asunercept and to obtain more clinical information for future asunercept application.

## Conclusions

In this study, we demonstrated that combined therapy of CCRT and 400 mg/week CAN008 significantly prolonged the OS of newly diagnosed GBM patients compared to patients with CCRT alone. A higher TMB in patients with a better CAN008 treatment response implies the involvement of CAN008 in regulating the activation of the GBM immune microenvironment and suggests that TMB is a predictive biomarker of the CAN008 treatment response. Asunercept is a potential targeted therapy drug when combined with current standard therapy in newly diagnosed GBM and when combined with reirradiation in recurrent GBM. Treatment with asunercept in GBM may not only alter tumor invasiveness but also the immune response, which requires more efforts to be clarified.

## Conflicts of interest

The authors declare the following financial interests/personal relationships which may be considered as potential competing interests: Kuo-Chen Wei reports financial support was provided by CANbridge Pharmaceuticals Inc. Gerald F. Cox and Fang-Min Huang report a relationship with CANbridge Pharmaceuticals Inc. that includes: employment.
