# Supplementary material for: CAN008 prolongs overall survival in patients with newly diagnosed GBM characterized by high tumor mutational burden
Source: Biomed J. 2023 Sep 21;47(4):100660. doi: 10.1016/j.bj.2023.100660 (PMC11340566; doi:10.1016/j.bj.2023.100660)
Supplement: Multimedia component 1 [file mmc1.docx]

**Supplementary data**

**Materials and Methods**

**Immunohistochemistry**

Tissue sections from the formalin-fixed, paraffin-embedded blocks were deparaffinized in xylene and rehydrated in a series of diluted alcohols. Tissue slides were then immersed in boiled Epitope Retrieval Solution pH 6 (Novocastra) for 10 minutes. Immunohistochemical staining was performed using the UltraVision Quanto Detection System HRP DAB (Thermo Fisher) according to the manufacturer’s protocol. Sections were incubated with a rabbit polyclonal antibody against PD-L1 (GTX104763, Genetex) or mouse monoclonal antibodies against PD-1 (ab52587, abcam) and CD5 (NCL-L-CD5-4C7, Leica) at 1:100 (PD-1 and CD5) or 1:500 (PD-L1) in 1% bovine serum albumin (BSA)/phosphate-buffered saline (PBS) overnight in 4°C. All sections were counterstained with hematoxylin (Sigma) and mounted with mounting medium. The protein expression was categorized into 4 groups (+0, +1, +2, and +3) according to the expression percentage and intensity in tumors.

**Supplementary Figures**


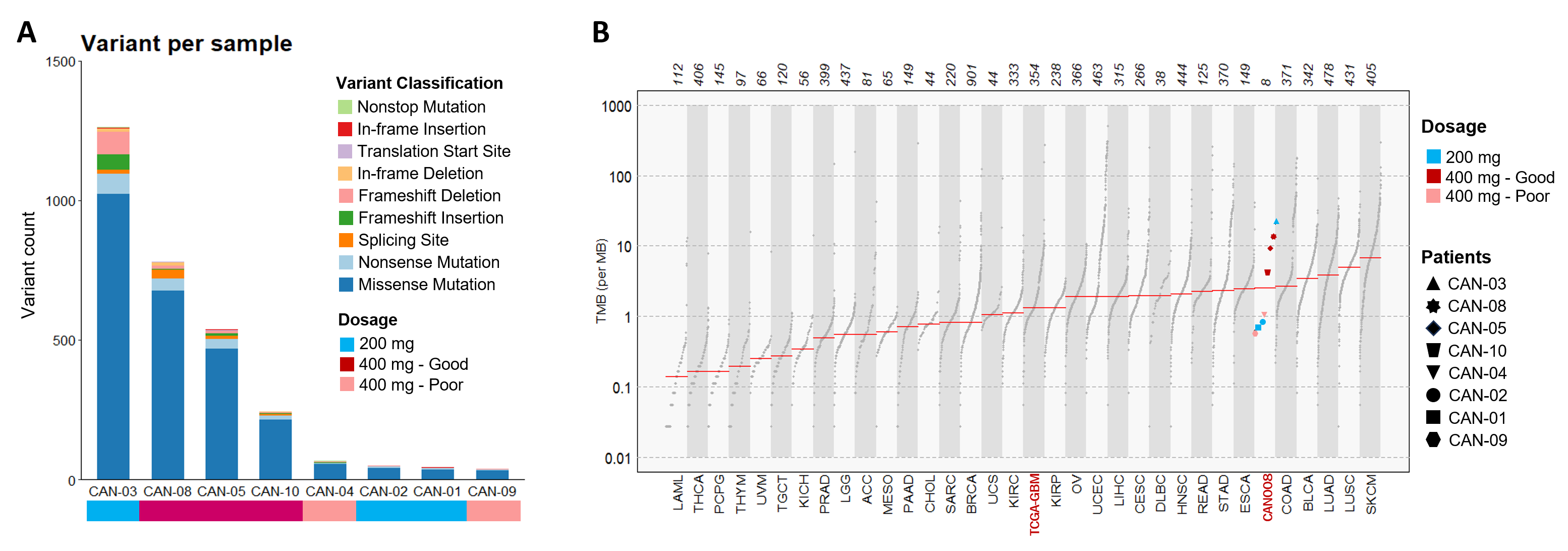


**Supplementary Figure 1.** Somatic mutation and TMB in the CAN008 patients treated with 200 or 400 mg/week. (A) Variant counts in each patient treated with CAN008. (B) Comparison of TMB measured in mutations per megabase (Mb) of DNA in the CAN008 treated patients and 33 TCGA datasets. Horizontal lines: median frequencies of TMB in each dataset.


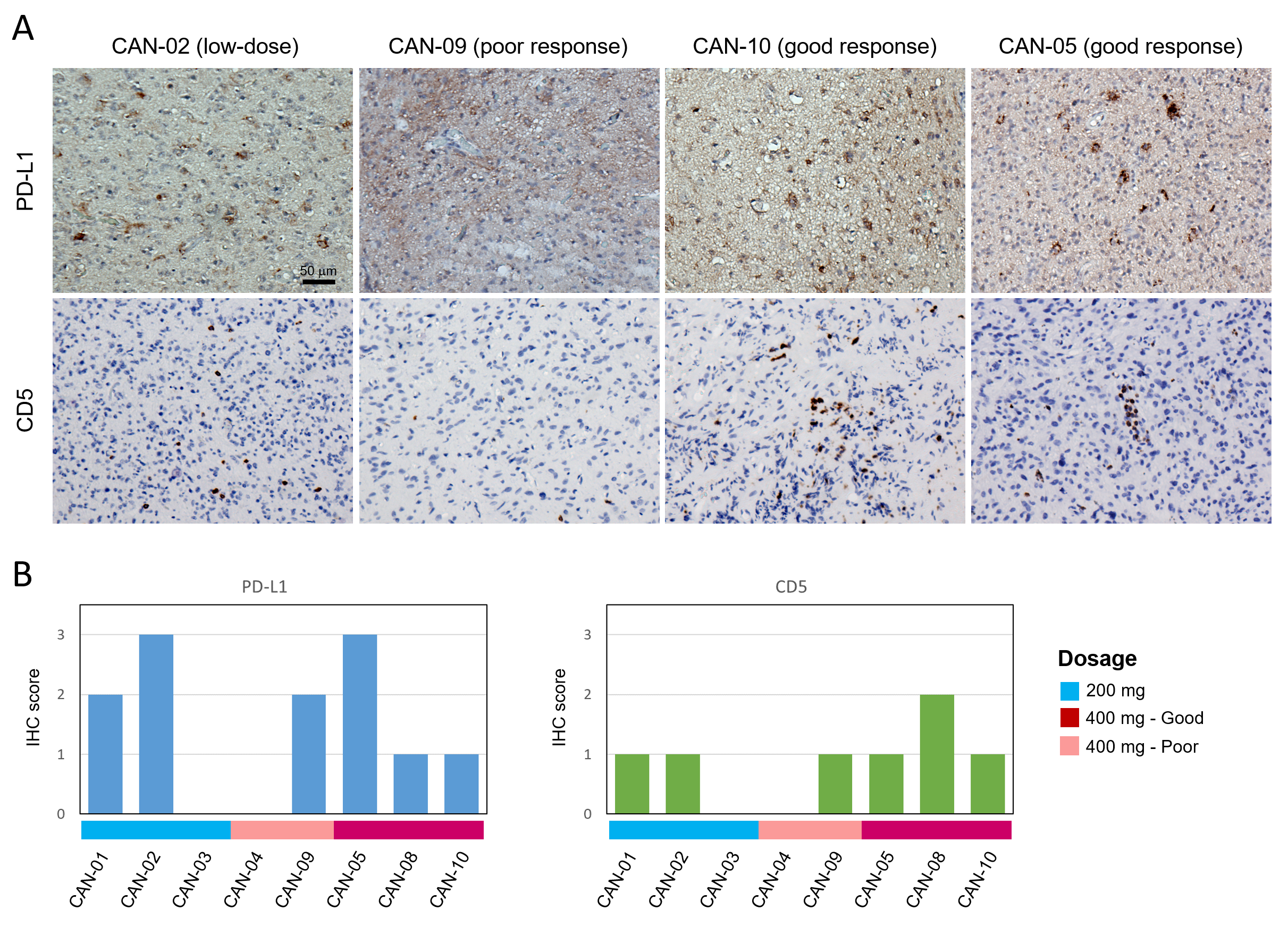


**Supplementary Figure 2.** Immunohistochemistry (IHC) staining of PD-L1 and CD5 expression in tumors. (A) Representative IHC staining images showed the PL-L1 and CD5 expression in tumors from CAN008 low-dose, poor response, and good response patients. (B) The IHC scores of PD-L1 and CD5 expression in each patient.
